# Computed Tomographic Findings of Eosinophilic Enteritis in Eight Cats: Case Series

**DOI:** 10.1002/vms3.70353

**Published:** 2025-04-26

**Authors:** Toshiyuki Tanaka, Hana Tsuruta, Koudai Furukawa, Hideo Akiyoshi

**Affiliations:** ^1^ Laboratory of Veterinary Advanced Diagnosis and Treatment, School of Veterinary Science Osaka Metropolitan University Osaka Japan; ^2^ Kinki Animal Medical Training Institute & Veterinary Clinic Osaka Japan; ^3^ Department of Veterinary Clinical Sciences, College of Veterinary Medicine Purdue University West Lafayette Indiana USA; ^4^ Japan Animal Referral Medical Center Osaka Osaka Japan; ^5^ Laboratory of Veterinary Surgery, School of Veterinary Science Osaka Metropolitan University Osaka Japan

**Keywords:** colon, duodenum, eosinophilic enteritis, feline, gastrointestinal eosinophilic sclerosing fibroplasia, lymphadenomegaly

## Abstract

In cats, eosinophilic enteritis (EE) is diagnosed when eosinophils are the predominant inflammatory cells on histopathology and no underlying trigger for their presence can be identified. Gastrointestinal eosinophilic sclerosing fibroplasia is a unique fibroblastic response of EE. Contrast‐enhanced computed tomography (CT) facilitates the objective characterisation of intestinal lesions and associated pathology. To the best of our knowledge, there are no reports of CT findings in cats with EE.

This case series retrospectively evaluated the CT findings of eight cats with EE including lesion location, intestinal wall layering structure, mass formation, location and size of lymph nodes, total wall thickness and outer intestinal layer relative thickness. The development of a layered intestinal wall appearance in the early and delayed phases was detected in seven (87.5%) and five cases (62.5%), respectively. All patients exhibited intestinal wall thickening, seven (87.5%) with marked thickening of the outer intestinal wall. Lesions were diffuse in all cats, involving the duodenum, jejunum and ileum in seven cats (87.5%) and in the ileum and colon in one cat (12.5%). Mass formation and lymphadenomegaly was detected in one (12.5%) and four cats (50%), respectively. The CT features of EE included thickening of the outer intestinal layer with development of a layered wall appearance and associated lymphadenomegaly.

## Graphical abstract

Eight cats with eosinophilic enteritis (EE) showed seven (87.5%) marked thickening of outer intestinal layer with layering structure (*) and four (50%) rounded lymphadenomegaly (**) and one (12.5%) mass formation (arrowhead), respectively. The intestinal wall thickening was observed in seven (87.5%) involving the duodenum, jejunum and ileum, in one (22.5%) involving the ileum and colon.

EE caused thickening of outer intestinal layer with layering structure and rounded lymphadenomegaly on computed tomography (CT).

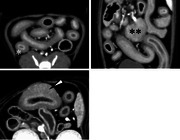

**Abbreviations**
CT: computed tomography
EE: eosinophilic enteritis
GI: gastrointestinal
GESF: gastrointestinal eosinophilic sclerosing fibroplasia
LA: long-axis
SA: short-axis

## 1 Introduction

In cats, eosinophilic enteritis (EE) is diagnosed when eosinophils are the predominant inflammatory cells on histopathology and no other triggers for the presence of eosinophils can be identified (Kleinschmidt et al. 2010; Griffin and Meunier 1990; Jergens 2012). EE is characterised by intestinal wall thickening primarily associated with thickening of the muscular layer (Tucker et al. 2014). In cats, gastrointestinal eosinophilic sclerosing fibroplasia (GESF) has been recently reported. GESF is a mass characterised by dense collagen trabeculae, large fibroblasts and numerous eosinophils (Craig et al. 2009). The aetiology of GESF is unknown, but it is hypothesised that cats with a genetic disposition develop GESF as a response to an external pathogen (Goffart et al. 2022; Černá et al. 2024).

In cats with EE, a mixture of the circumscribed and diffuse type has been reported based on pathology (Hendrick 1981). A previous study on GESF reported that three of four cases showed intestinal wall thickening adjacent to the mass (Weissman et al. 2013). One study proposed that GESF is a unique fibroblastic response of EE (Craig et al. 2009).

Computed tomography (CT) is a widely performed technique that can objectively characterise lesions via contrast enhancement, aiding in determination of the lesion's site, appearance and detecting local invasion (Lee et al. 2010). There is limited information on CT findings regarding the cat's intestinal tract but in a normal cat, the layering structure of the intestinal wall is obscured on contrast CT (Holle et al. 2023). Although CT is used to investigate a limited number of intestinal diseases, there are no reports of CT findings in cats with EE. This case series aimed to retrospectively evaluate the CT findings of EE in eight cats.

## 2 Materials and Methods

This study was a retrospective case series. We reviewed the records of all cats with chronic gastrointestinal (GI) signs that underwent CT and histopathological examinations at Osaka Metropolitan University and Kinki Animal Medical Training Institute and Veterinary Clinic between 2014 and 2023. The inclusion criterion was the presence of concurrent histopathologically diagnosed EE. The exclusion criterion was tumour development.

Whole‐body CT studies were performed using a multidetector 16‐slice CT scanner (SOMATOM Scope; SIEMENS, Tokyo, Japan or Activion16; Canon Medical Systems Corporation, Tochigi, Japan) in the helical scan mode according to our previous protocol (Tanaka et al. 2019). All cats were induced with 7 mg/kg of propofol (Propofol 1%; MSD Animal Health K.K., Tokyo, Japan) and maintained with isoflurane (2%) and oxygen with an endotracheal tube. For contrast‐enhanced imaging, all cats were administered 2 mL/kg (300 mgI/mL) of the non‐ionic contrast medium, iohexol (Ioverin 300; Teva Pharma Japan, Inc., Aichi, Japan) via an indwelling intravenous cannula placed in the cephalic vein. The injection duration was 20 s. A contrast‐enhanced study was performed on the abdomen in the early (20 s after the injection of contrast medium) and delayed phases (60 s). Image analyses were performed according to our previous report using a commercially available DICOM image viewing software (OsiriX 13.0.1, 64 bit, Pixmeo, Switzerland; Tanaka et al. 2019). All images were reviewed by two experienced veterinary radiologists, and the CT features were determined by consensus. Images were assessed in random order in three different readout sessions with at least 2‐week intervals between each session to minimise potential bias.

The following CT features were recorded: qualitative parameters including the development of a layered intestinal wall appearance in the early and delayed contrast phases (presence or absence), single or diffuse lesions, mass formation (presence or absence), lymphadenomegaly (presence or absence) and location of lymphadenomegaly. Quantitative parameters included the maximum short‐axis (SA) diameter, maximum long‐axis (LA) diameter and the short‐axis diameter to long‐axis diameter ratio (SA/LA) of enlarged lymph node, total wall thickness and the relative thickness of the outer intestinal layer. Layering structure and wall thickness were assessed in areas where the intestinal lumen was not distended by gas.

Development of a layered wall structure was considered to be present if delineation of the intestinal mucosa and outer wall was evident in the post‐contrast images. According to the previous literature (Holle et al. 2023), we defined the high attenuation area in the early and late phase as the mucosal layer and the lower attenuation area as the outer layer of the intestinal wall, including the muscular and serosal layer. Mass formation was defined as the presence of asymmetrical intestinal wall thickening. Lymphadenomegaly was recorded when the lymph node length or width was longer than the mean normal size (Perlini et al. 2018; Smith et al. 2020). SA (mm) and LA diameters (mm) of the enlarged lymph nodes were measured in any reformatted plane (transverse, sagittal or dorsal) as described in a previous study (Tanaka et al. 2019). SA/LA was also calculated. Total wall thickness was defined by measuring the distance from the luminal side of the mucosa to the outer surface of the serosa at the site of the lesion. In the case of diffuse lesions, a minimum of three measurements were taken and the average was reported. Where the development of a layered wall appearance was evident, the thickness of the outer intestinal layer was also recorded. Based on previous literature (Tucker et al. 2014), relative wall thickness of the outer intestinal layer was defined as the thickness of the outer intestinal layer as a percentage of the total intestinal wall thickness (Donato et al. 2014).

## 3 Results

CT imaging studies of 92 cats with chronic GI signs were identified. Of these, 30 cats were excluded due to a diagnosis of neoplasia including lymphoma, adenocarcinoma and leiomyoma. Of the remaining 62 cats, eight had histopathologically diagnosed EE and met the final criteria for inclusion in the analyses. All cats were initially sampled endoscopically, identifying four cats (50%) with eosinophilic inflammation in the duodenum and one cat (12.5%) with eosinophilic inflammation in the colon. Following inconclusive endoscopic histopathology results, three cats (37.5%) underwent laparotomy and were diagnosed with EE based on full thickness ileal biopsies.

The cats comprised two neutered males, one intact male and five spayed females. The mean (± SD) age was 7.9 ± 3.1 years. Five cats were mixed breed and the remaining three were Somali, American Shorthair and Singapura breeds.

Development of a layered intestinal wall appearance was detected in the early and delayed contrast phases in seven (87.5%) and five cats (62.5%), respectively. Diffuse lesions were detected in all eight cats; seven (87.5%) with involvement of the duodenum, jejunum and ileum, and one (12.5%) with involvement of the ileum and colon. Mass formation, within the outer layer of the jejunal EE region, was detected in one cat (12.5%), fine needle aspiration cytology of which revealed eosinophils.

**FIGURE 1:**
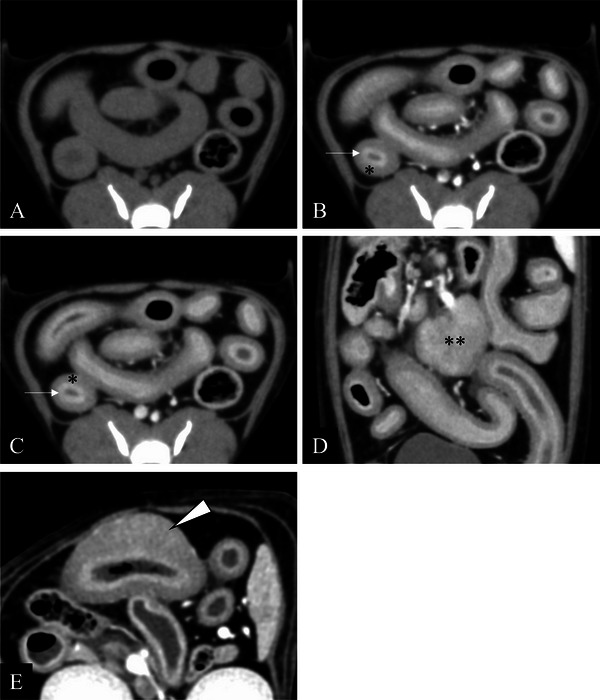
Representative computed tomography (CT) images of eosinophilic enteritis (EE) in pre‐contrast (A), early‐phase post‐contrast (B), delayed‐phase post‐contrast (C) and reformatted plane (D). EE showed thickening of outer intestinal layer with development of a layered wall appearance and rounded lymphadenomegaly. In one cat, the mass formed (arrowhead) in the outer layer of the jejunal EE region (E). Mucosal layer (arrow), outer intestinal layer (*), enlarged lymph node (**).

Lymphadenomegaly was detected in four cats (50%), one with colic, two with jejunal and one with sternal lymph node enlargement. Total wall thickness was above the reported normal limit in all eight cats with an average (mean ± SD) thickness of 4.5 ± 2.2 mm (2.0 mm, 1.9 mm, 1.8 mm and 1.0 mm being considered normal for duodenal, jejunal, ileal and colonic wall thicknesses, respectively [Holle et al. 2023]). The signalment and predominant CT features of the eight cats with EE are summarised in Table 1. Additional CT findings included ascites in two cats (25%). Representative images of the prevalent CT findings found in the eight cats with EE are shown in Figure 1.

**TABLE 1:** The clinical findings and computed tomography (CT) features of eight cats with eosinophilic enteritis (EE).

			Layered intestinal wall appearance									
Breed	Age (years)	Sex	Early	Delayed	Total wall thickness^b^ (mm)	Relative thickness^c^ (%)	Lesion locations^d^	Mass formation	Lymph adenomegaly	LA (mm)	SA (mm)	SA/LA
Somali	7	MN	−	−	2.85	N/A	d, j, i	−	−	N/A	N/A	N/A
Mixed	11	FS	+	−	3.0	47	d, j, i	−	Sternal	21	18	0.86
Mixed	4	ME	+	+	4.7	53	d, j, i	−	−	N/A	N/A	N/A
Mixed	4	FS	+	+	9.2	61	i, c	−	Colic	15	13	0.87
Mixed^a^	13	MN	+	+	3.9	62	d, j, i	−	−	N/A	N/A	N/A
American Shorthair	9	FS	+	+	5.4	50	d, j, i	−	Jejunal	27	25	0.93
Singapura	7	FS	+	−	2.1	71	d, j, i	−	−	N/A	N/A	N/A
Mixed^a^	8	FS	+	+	4.6	80	d, j, i	+	Jejunal	67	35	0.52

Abbreviations: LA, long axis; SA, short axis.

^a^ Cats with ascites.

MN, cast male; ME, intact male; FS, spayed female.

^b^ Reported mean normal intestinal wall thickness: duodenum 2.0 mm, jejunum 1.9 mm, ileum 1.8 mm and colon 1.0 mm (Holle et al. 2023).

^c^ Relative thickness: thickness of the outer intestinal layer/the total intestinal wall thickness.

^d^ Detected segment of lesion; d, duodenum; j, jejunum; i, ileum; c, colon.

## 4 Discussion

In cats, EE is characterised by marked thickening of the intestinal muscularis layer and peripheral eosinophilia (Tucker et al. 2014). In humans with EE, it has been shown that secretory products of eosinophils, cytokines and chemokines including interleukin (IL)‐13, IL‐4, IL‐5, eotaxin, and transforming growth factor‐beta on smooth muscle are responsible for the muscularis thickening (Cheng et al. 2012). This wall thickening, particularly affecting the muscularis layer, can clearly be demonstrated on ultrasonography (Tucker et al. 2014). In the cats reported here, the development of an abdominal layered appearance with thickening of the outer intestinal layer appeared to be a consistent CT finding. Similar changes of wall thickening and layering of the intestinal wall are noted in human with EE, but this is often accompanied by diffuse mucosal fold thickening and luminal narrowing with or without intestinal obstruction, which was not a feature in the eight cats described here (Horton et al. 1999; Vitellas et al. 1995; Zheng et al. 2008; Wiesner et al. 2002; Anuradha et al. 2012). Layering of the intestinal wall and diffuse mucosal fold thickening are observed in many inflammatory intestinal lesions, and are not confined to EE (Anuradha et al. 2012). In contrast, this study suggests that thickening of the outer intestinal layer on CT may be helpful to differentiate EE from other inflammatory intestinal pathologies in cats, however further studies are needed.

In the normal duodenum and ileum, mucosal enhancement is detected in the early and late CT contrast phases (Holle et al. 2023). The villi on the mucosal surface of the normal intestine have a single arteriolar loop projecting into each individual villus, which connects to the submucosal venule (Fitzgerald et al. 2017). This results in mucosal enhancement in the early and late CT contrast phases (Kvietys 2010). In the eight cats reported here, all had diffuse alteration of the intestinal layering structure and thickening of the outer intestinal layer and, in one cat, a mass was apparent. On ultrasonography, GESF usually appears as focal masses with symmetrical or asymmetrical circumferential wall thickening, loss of layering structure and a necrotic centre (Černá et al. 2024; Weissman et al. 2013). However, in 20% of the cases, an altered layering structure is observed (Černá et al. 2024). In the cat reported here, the mass was not surgically removed and histopathological examination was not performed, thus, its relationship to GESF is unknown. However, the formation of mass lesions as well as diffuse lesions in cats with EE has been proposed (Weissman et al. 2013). Further studies are needed for better comprehension of the relationship between EE and GESF.

In humans, eosinophilic GI disorders are diagnosed based on the histologic findings of eosinophilic infiltration of the GI wall (Klein et al. 1970) and they are classified into three patterns depending on the primary site of involvement: mucosa, muscle and serosa (Talley et al. 1990). Symptoms vary depending on the affected site and layers of the lesion, with serous lesions producing ascites (Talley et al. 1990). In this study, two of the eight cats had ascites, suggesting the possibility of serosal invasion.

Abdominal lymph nodes were assessed according to size, shape, attenuation, enhancement based on previous descriptions and feline reference ranges (Tobón Restrepo et al. 2022; Schreurs et al. 2008; Griffin 2021). Enlarged lymph nodes were documented in four of the eight cats with EE and all displayed a rounded appearance. It is notable that, in one cat, the sternal lymph node was enlarged in the absence of abdominal lymphadenopathy. In cats, non‐regional lymph node enlargement has been reported in EE with peripheral eosinophilia and multivisceral infiltration of eosinophils (Hendrick 1981). The presence of peripheral eosinophilia and visceral infiltration of eosinophils was not investigated in this study.

This study has some limitations. Most importantly, is that the intestinal lesions were determined based on CT findings without concurrent ultrasonographic comparison, and without histopathological confirmation of eosinophilic inflammation in all abnormal areas. Further studies are needed to evaluate the relationship between lymphadenomegaly, peripheral eosinophilia and CT findings in a large population of cats with EE. In conclusion, in cats with EE, thickening of the outer intestinal layer with development of layered small intestinal wall appearance and rounded lymphadenomegaly appear to be common findings on CT imaging.

## Author Contributions

Toshiyuki Tanaka was the principal investigator and first author of the manuscript. Toshiyuki Tanaka conceived the idea of the study. Hideo Akiyoshi supervised the surveillance components. Toshiyuki Tanaka, Hana Tsuruta and Koudai Furukawa validated, analysed and interpreted the data. Toshiyuki Tanaka prepared the initial draft, figures and table. All the authors contributed to the writing and editing of the manuscript.

## Ethics Statement

The owners of the cats described in this study provided informed consent for the diagnostic procedures, treatment and use of clinical data such as medical history, imaging studies and histopathological findings for research and publication purposes. Since all diagnostic studies and initiated treatments were part of daily clinical activities, the study did not reach the threshold for submission to the local ethics and welfare committee.

## Conflicts of Interest

The authors declare no conflicts of interest.

### Peer Review

The peer review history for this article is available at https://www.webofscience.com/api/gateway/wos/peer‐review/10.1002/vms3.70353.

## Data Availability

The data that support the findings of this study are available from the first author, Toshiyuki Tanaka, upon reasonable request.
